# Determinants of the creatinine clearance to glomerular filtration rate ratio in patients with chronic kidney disease: a cross-sectional study

**DOI:** 10.1186/1471-2369-14-268

**Published:** 2013-12-05

**Authors:** Yen-chung Lin, Nisha Bansal, Eric Vittinghoff, Alan S Go, Chi-yuan Hsu

**Affiliations:** 1 Division of Nephrology, Department of Internal Medicine, Taipei Medical University Hospital, Taipei, Taiwan; 2 Department of Internal Medicine, School of Medicine, College of Medicine, Taipei Medical University, Taipei, Taiwan; 3 Division of Nephrology, School of Medicine, University of California-San Francisco, San Francisco, CA, USA; 4 Department of Epidemiology and Biostatistics, School of Medicine, University of California-San Francisco, San Francisco, CA, USA; 5 Division of Research, Kaiser Permanente Northern California, Oakland, CA, USA

**Keywords:** Albuminuria, Chronic kidney disease, Glomerular filtration rate, Race/Ethnicity

## Abstract

**Background:**

Creatinine secretion, as quantified by the ratio of creatinine clearance (CrCl) to glomerular filtration rate (GFR), may introduce another source of error when using serum creatinine concentration to estimate GFR. Few studies have examined determinants of the CrCl/GFR ratio. We sought to study whether higher levels of albuminuria would be associated with higher, and being non-Hispanic black with lower, CrCl/GFR ratio.

**Methods:**

We did a cross-sectional analysis of 1342 patients with chronic kidney disease from the Chronic Renal Insufficiency Cohort (CRIC) who had baseline measure of iothalamate GFR (iGFR) and 24-hour urine collections. Our predictors included urine albumin as determined from 24-hour urine collections (categorized as: <30, 30-299, 300-2999 and ≥3000 mg), and race/ethnicity (non-Hispanic white, non-Hispanic black, Hispanic). Our outcome was CrCl/iGFR ratio, a measure of creatinine secretion.

**Results:**

Mean iGFR was 48.0 ± 19.9 mL/min/1.73 m^2^, median albuminuria was 84 mg per day, and 36.8% of the study participants were non-Hispanic black. Mean CrCl/iGFR ratio was 1.19 ± 0.48. There was no association between the CrCl/iGFR ratio and urine albumin (coefficient 0.11 [95% CI−0.01-0.22] for higest verus lowest levels of albuminuria, p = 0.07). Also, there was no association between race/ethnicity and CrCl/iGFR ratio (coefficient for non-Hispanic blacks was−0.03 [95% CI−0.09-0.03] compared with whites, p = 0.38).

**Conclusions:**

Contrary to what had been suggested by prior smaller studies, CrCl/GFR ratio does not vary with degree of proteinuria or race/ethnicity. The ratio is also closer to 1.0 than reported by several frequently cited reports in the literature.

## Background

In clinical practice and research studies, kidney function is most often estimated using concentration of serum creatinine, an endogenous filtration marker. This is based on the assumption that creatinine clearance (CrCl) approximates GFR. In considering the limitations of serum creatinine-based estimates of glomerula filtration rate (GFR), much attention has been given to problems stemming from variations in creatinine production which may be reduced as a result of factors such as malnutrition, advanced age or liver disease. Less attention has been given to the fact that creatinine is cleared by the kidneys not only by filtration but also by tubular secretion. So variations in creatinine secretion may introduce another source of error when using serum creatinine concentration to estimate kidney function.

Prior research has shown or suggested that several factors influence the rate of tubular secretion of creatinine which can be quantified as the CrCl/GFR ratio [1]. CrCl/GFR ratio increases as GFR decreases [2,3] (i.e. tubular secretion plays an increasing important role in renal excretion of creatinine as GFR falls). Other studies have reported that patients with high levels of proteinuria may have high CrCl/GFR ratio. For example, one study reported that patients with nephrotic range proteinuria and low serum albumin had higher tubular secretion of creatinine of 36 ml/min/1.73 m^2^ (vs. 24 ml/min/1.73 m^2^ for those with less severe nephrotic syndrome) [4]. Finally, some studies have suggested that African-American patients may have lower CrCl/GFR ratio than non-African Americans [5,6].

However, these prior studies of the relation between CrCl and GFR have been limited by small sample sizes and inclusion of only a limited spectrum of kidney disease (such as only patients with glomerular disease) [7], and lack of calibration of serum creatinine measurements which render results harder to interpret as systemic errors in serum creatinine measurements may lead to higher or lower CrCl values [6]. In addition, some studies quantified urine protein via excretion of total protein rather than albumin. Measurement of total urine protein is not possible to standardize and is increasingly considered a suboptimal meter of renal dysfunction compared with measurement of albuminuria [8,9].

To address these knowledge gaps, we explored factors which influence CrCl/GFR ratio in a large diverse cohort of patients with chronic kidney disease (CKD), who had calibrated serum creatinine measurements and quantification of 24-hour urine albumin [10,11]. We *a priori* wanted to examine whether greater degree of albuminuria was associated with higher and being non-Hispanic black with lower CrCl/GFR ratio.

## Methods

### Study population

We used baseline data from a subset of participants in the Chronic Renal Insufficiency Cohort (CRIC) study. CRIC is a multicenter prospective cohort sponsored by the National Institutes of Diabetes, Digestive and Kidney Disease (NIDDK) that enrolled patients from seven clinical centers throughout the United States. The design and baseline characteristics of the CRIC cohort (including rationale for inclusion and exclusion criteria) have been published [10,12]. Briefly, men and women between the ages of 21 and 74 were eligible for the study if they had reduced estimated GFR, based on Modification of Diet in Renal Disease (MDRD) study equation. Inclusion criteria were estimated GFR 20-70 ml/min per 1.73 m^2^ for person aged 21-44 years, 20-60 ml/min per 1.73 m^2^ for persons aged 45-64 years, and 20-50 ml/min per 1.73 m^2^ persons aged 65-74 years. Exclusion criteria included prior renal transplantation, polycystic kidney disease, multiple myeloma, recent use of immunosuppression, and severe comorbid illnesses, such as cirrhosis, HIV disease, and severe (New York Heart Association class III or IV) heart failure. A weighted random sample of approximately one third of the cohort (referred to as the subcohort) was assigned to undergo additional, more intensive testing, including ^125^I-iothalamate clearance studies to measure GFR. Enrollment started July 2003 and ended March 2007. Additional enrollment of Hispanic participants continued through August 2008 in one center (“Hispanic CRIC”). Of the 3939 participants in CRIC, we included in our study only the 1423 patients who participated in sub-study of ^125^I-iothalamate clearance. After excluding 81 enrollees who were missing 24-hour urine albumin, our final study sample was 1342.

### Measures of kidney function

Our study sample underwent direct GFR measurement by urinary clearance of ^125^I-iothalamate (iGFR). iGFR was conducted using a protocol similar to that in prior studies [11,13]. Briefly, after a water load and administration of saturated solution of potassium iodine (SSKI), ^125^I-iothalamate was injected subcutaneously. After a 60-to 90-min waiting period, timed collections of urine and serum were performed. Urine flow rate was maintained above 1 ml/min. The goal was to obtain four timed urine collection periods bracketed by blood draws to measure plasma iothalamate levels (P). Concurrent urine counts (U) and urine volumes (V) for each period were determined. GFR was calculated as weighted average UV/P and corrected for body surface area. In CRIC, 88% of subcohort enrollees had four or more urine collection periods, 6% had three, and 5% had two or fewer. Due to increased precision of measured GFR values with exclusion of the first clearance period, this measure was used as the reference standard in the analysis. The median coefficient of variation (CV) for the iGFR was 9.7%, excluding the first period [14].

Serum creatinine measurements were done in the CRIC central laboratory at University of Pennsylvania on the Hitachi Vitros 950 calibrated to the MDRD central laboratory at Cleveland Clinic [15] (because the original 4-variable MDRD equation [16] was used as the entry criteria for CRIC). For the analyses in this paper, we further calibrated the Cleveland Clinic creatinine to the standardized IDMS-traceable serum creatinine value as Standardized Cr = Cleveland Clinic serum creatinine*0.95 [17].

At baseline, urine creatinine and albumin excretion were determined from a 24-hour urine collection. Urine albumin was determined by immunoturbidometric assay (Roche Diagnostics) and urine creatinine was done spectrophotometrically with Jaffe method (Roche Diagnostics). The samples were rejected and re-collection attempted if total urine volumes were below 500 cc or collection times below 22 hours or more than 26 hours. All creatinine clearances were calculated in this paper as UV/P with standardized serum creatinine measurements.

### Assessments of predictors

We identified *a priori* several predictors of interest: 24-hour urine albumin, and self-reported race/ethnicity (non-Hispanic white, non-Hispanic black, Hispanic) defined at the baseline CRIC visit. We classified 24-hr urine albumin excretion as < 30 mg, 30-299 mg, and ≧300 mg [9]. To allow for the detection of an effect in the nephrotic range, we further divided the last group, macro-albuminuria (also known as severely increased albuminuria), in to above or below 3000 mg.

### Assessments of outcome

The outcome variable of our study was the CrCl to iGFR ratio. CrCl and iGFR were both expressed as ml/min normalized to body surface area.

### Statistical analysis

Baseline characteristics of CRIC subcohort participants were described using mean ± standard deviation or median (25th-75th percentiles) for continuous variables and number (percentage) for categorical variables.

Linear regression was used to test univariate associations between the CrCl/iGFR ratio and a comprehensive list of baseline demographic and clinical characteristics (Table 1). Characteristics that were found to be significantly associated with CrCl/iGFR ratio were included in subsequent multivariable models.

**Table 1:** Characteristics of the study population (N = 1342)

Creatinine clearance (CrCl) (mL/min/1.73 m^2^)	54.9 ± 27.2
Iothalamate measured glomerular filtration rate (iGFR) (mL/min/1.73 m^2^)	48.0 ± 19.9
CrCl/iGFR, mean ± SD	1.19 ± 0.48
CrCl/iGFR, median (25th-75th percentile)	1.15 (0.92-1.39)
24-hrs urine albumin (mg/24 hrs) [median (25th-75th percentile)]	84 (11-643)
Age (years)	56.2 ± 12.2
Male, %	56.7%
Physical examinations	
Body mass index (kg/m^2^)	31.3 ± 6.8
Body surface area (m^2^)	2.0 ± 0.3
Systolic blood pressure (mmHg)	129 ± 22
Diastolic blood pressure (mmHg)	73 ± 13
Race/Ethnicity; %	
Non-Hispanic white	42.3%
Non-Hispanic black	36.8%
Hispanic	14.0%
Others	6.9%
Self-reported comorbidities, %	
Diabetes	48.3%
Cardiovascular disease	26.4%
Peripheral vascular disease	5.7%
Stroke	8.0%
Current smoking, %	11.2%
Self-reported medication use, %	
Beta-blocker	46.9%
Loop diuretics	35.3%
Thiazide diuretics	27.9%
Angiotension receptor blockers or angiotensin-converting enzyme	68.6%
Statins	54.2%
Laboratory results	
Hematocrit (%)	37.4 ± 4.9
Albumin (g/dL)	3.9 ± 0.5
Serum creatinine (mg/dL)	1.61 ± 0.53
BUN (mg/dL)	29 ± 13
Triglyceride (mg/dL)	155 ± 112
LDL cholesterol (mg/dL)	103 ± 36
Fasting glucose (mg/dL)	113 ± 49
Hemoglobin A1C (%)	6.6 ± 1.6
hs-CRP (mg/L) [median (25th-75th percentile)]	2.2 (0.9-5.2)

We explored the distribution of CrCl/iGFR ratio and CrCl by quintiles of iGFR. Linear regression was to determine the association between CrCl/iGFR ratio and 24-hour urine albumin and race/ethnicity in crude and adjusted models. Because iGFR is in the denominator of the outcome, we did not include it as a predictor.

In a sensitivity, analysis to reduce the skewness of residuals, we examined log-transformed CrCl/iGFR ratio as the outcome of interest.

All statistical analyses were performed using STATA 12 (STATA Corp, College Station, TX). A p-value < 0.05 was considered statistically significant.

### Regulatory approval

De-identified data for this analysis were retrieved from the National Institutes of Diabetes and Digestive and Kidney Disease (NIDDK) Data Repository (https://www.niddkrepository.org/niddk/home.doH) after appropriate institutional review board approval was obtained (University of California San Francisco Committee on Human Research IRB Number: 10-04231).

## Results

Among the 1,342 participants in our study, the mean age was 56 years, 57% were men, and 37% were non-Hispanic black. The mean (± standard deviation [SD]) CrCl was 54.9 ± 27.2 mL/min/1.73 m^2^ and mean iGFR 48.0 ± 19.9 mL/min/1.73 m^2^ (Table 1). Mean CrCl/iGFR ratio was 1.19 ± 0.47 and median CrCl/iGFR ratio was 1.15 (with interquarile range [IQR] 0.92-1.39). The median time lapse between 24-hour urine collection and iGFR measurement was 0 days (IQR 0 to 11 days) (mean time lapse 21 days).

We found, similar to prior studies, that patients with lower iGFR had higher CrCl/iGFR ratios (Table 2).

**Table 2:** Creatinine clearance (CrCl), CrCl/iGFR, classified by quintiles of iothalamate-125 glomerular filtration rate (iGFR) (N = 1342)

**Quintiles of iGFR (ml/s/1.73 m**^**2**^**)**	**iGFR (ml/min/1.73 m**^**2**^**), median (IQR)**	**CrCl (ml/min/1.73 m**^**2**^**), median (IQR)**	**CrCl/iGFR, median (IQR)**
1 (N = 269)	24.8 (20.8—27.4)	31.7 (25.3—39.3)	1.33 (1.06—1.62)
2 (N = 268)	35.7 (33.0—38.5)	42.4 (33.6—51.4)	1.20 (0.94—1.47)
3 (N = 269)	45.7 (43.3—48.0)	52.5 (41.2—61.3)	1.14 (0.92—1.33)
4 (N = 268)	56.3 (53.1—59.8)	61.9 (50.1—73.1)	1.10 (0.87—1.28)
5 (N = 268)	74.7 (68.4—84.9)	81.9 (65.0—96.6)	1.05 (0.86—1.22)

Of the characteristics listed in Table 1, CKD patients with higher BUN, high creatinine and lower hemoglobin had higher CrCl/iGFR ratio. The only other factors associated with the CrCl/iGFR ratio were the use of loop diuretics (associated with higher CrCl/iGFR ratio of 0.09, p = 0.001) and hemoglobin A1C (each 1% increase in hemoglobin A1c was associated with a 0.02 lower CrCl/iGFR ratio, p = 0.007). There was no association with use of thiazide diuretics.

**Figure 1:**
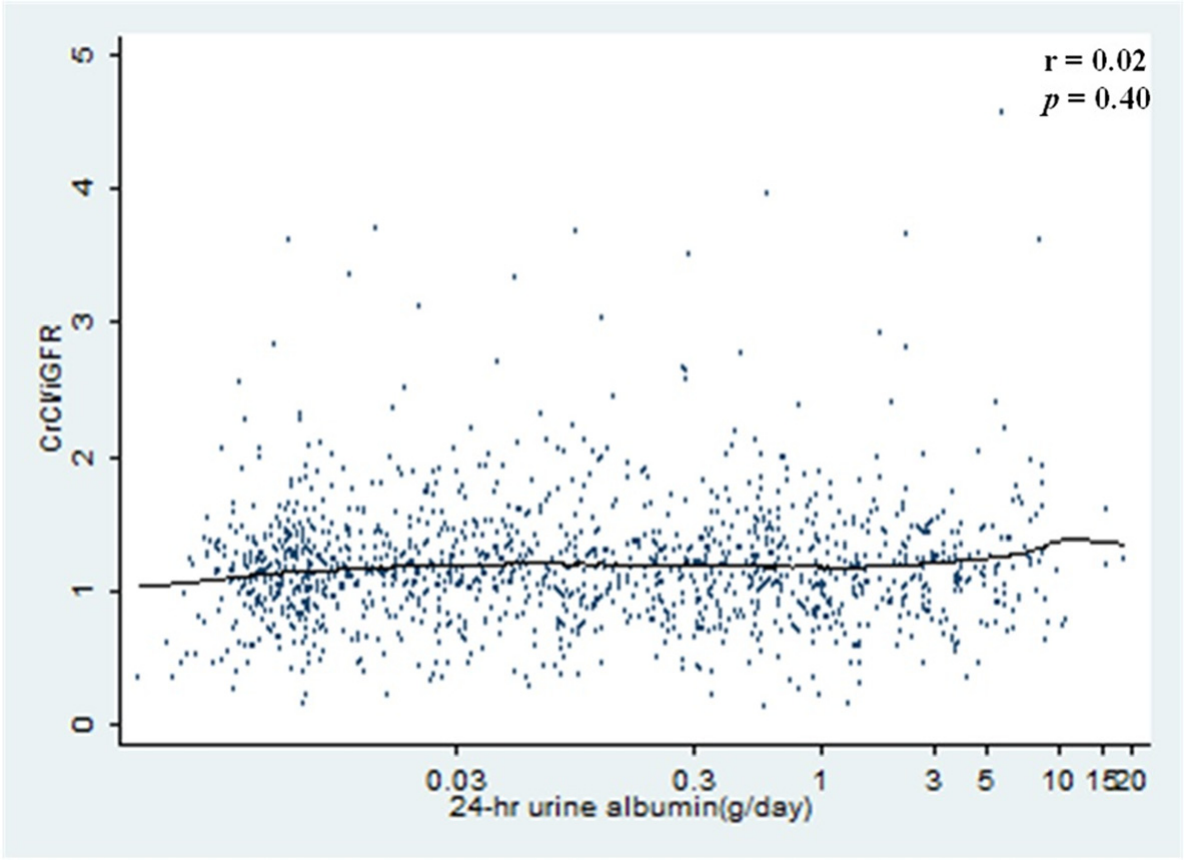
**Scatter plot with a locally weighted scatterplot smoothing line showing that 24-hrs urinary albumin is not correlated with CrCl/iGFR (rs = 0.02, *P*** **= 0.40 by Spearman’s correlation test) (two outlier with CrCl/iGFR ratio of 6.71, 0.04 were omitted).**

There was no significant correlation between degree of albuminuria and ratio of CrCl/iGFR (rs = 0.02, *p* = 0.40 in Spearman’s correlation test) (Figure 1). In unadjusted models, categories of albuminuria were not associated with higher CrCl/iGFR ratios (Table 3). Results were unchanged in multivariable analyses controlling for age, sex, race/ethnicity, use of loop diuretics and hemoglobin A1c levels (Table 3). Even patients with ≥ 3000 mg of 24-hour urine albumin did not appear to have increased tubular secretion of creatinine compared to normal albuminuria group. Additionally, CrCl/iGFR ratio in patients (N = 47) with serum albumin <3.0 g/dl was 1.21 ± 0.48 and that in with serum albumin ≥3.0 g/dl (N = 1260) was 1.19 ± 0.48 (p-value 0.89). Also there was no association between degree of albuminuria and the ratio of CrCl/iGFR within substrata of iGFR level (data not shown).

**Table 3:** The association of 24-hrs urinary albumin in categorical classifications and CrCl/iGFR ratio in the regression model (N = 1342)

	**Normal (24-hrs urine albumin < 30 mg; N = 515)**	**Microalbuminuria (24-hrs urine albumin 30 to** ≦**299 mg; N = 343)**	**Macroalbuminuria (24-hrs urine albumin 300 to** ≦**2999 mg; N = 378)**	**Nephrotic-range proteinuria (24-hrs urine albumin** ≧**3000 mg; N = 106)**
Absolute change in CrCl/iGFR (95% CI)				
Unadjusted	Reference	0.05 (−0.01—0.12)	0.02 (−0.04—0.09)	0.07 (−0.04—0.17)
		*P* = 0.12	*P* = 0.48	*P* = 0.20
Multivariate adjusted^a^	Reference	0.06 (−0.01—0.13)	0.05 (−0.03—0.12)	0.11 (−0.01—0.22)
		*P* = 0.08	*P* = 0.21	*P* = 0.07

CrCl = Creatinine clearance; IGFR = I^125^ Iothalamate measured glomerular filtration rate.

^a^Adjusted for age, sex, race/ethnicity, use of loop diuretics, hemoglobin A1C levels.

Mean CrCl/iGFR was not significantly different between non-Hispanic black and non-Hispanic white patients (Table 4). The results were similar after further adjusting for age, sex, race/ethnicity, use of loop diuretics and hemoglobin A1c levels. CRIC participants who were Hispanic also did not have statistically different CrCl/iGFR ratios compared with non-Hispanic white participants (Table 4). There was no association between race/ethnicity and the ratio of CrCl/iGFR within substrata of iGFR level.

**Table 4:** **CrCl = creatinine clearance; IGFR = I**^**125 **^**Iothalamate measured glomerular filtration rate**

	**Non-Hispanic white**	**Non-Hispanic black**	**Hispanics**	**Others**
	**(N = 568)**	**(N = 494)**	**(N = 188)**	**(N = 92)**
Absolute change in CrCl/iGFR ratio (95% CI)				
Unadjusted	Reference	−0.03 (−0.09—0.03)	0.01 (−0.07—0.09)	−0.01 (−0.12—0.09)
		*P* = 0.34	*P* = 0.89	*P* = 0.79
Multivariate adjusted^a^	Reference	−0.03 (−0.09—0.03)	0.02 (−0.07—0.10)	0.01 (−0.10—0.11)
		*P* = 0.38	*P* = 0.72	*P* = 0.91

CrCl = Creatinine clearance; iGFR = I^125^ Iothalamate measured glomerular filtration rate.

Others = American Indian/Alaskan Native, Asian/Asian American, or Native Hawaiian/Other Pacific islander.

^a^Adjusted for age, sex, use of loop diuretics, hemoglobin A1C.

Similar results were seen in a sensitivity analysis using log-transformed CrCl/iGFR ratio as the outcome (data not shown).

## Discussion and conclusions

In this well characterized cohort of CKD patients with mean measured GFR of 48 m/min/1.73 m^2^, we found that the mean CrCl/GFR ratio was 1.19. This ratio is considerably lower than that reported by older papers in the literature (although similar to certain more recently published studies [18]). For example, in a frequently cited article based on 171 patients, Shemesh et al. reported that in the range of measured GFR of 40-80 ml/min/1.73 m^2^ (mean 60 ml/min/1.73 m^2^) the CrCl/GFR ratio was 1.57 [7,19,20]. Bauer et al. described CrCl/GFR ratios of 1.62-1.87 when measured GFR was 40-70 ml/min/1.73 m^2^[3]. Reasons for this potential difference may include the fact that both Shemesh and Bauer measured GFR using inulin clearance rather than iothalamate clearance and the former is known to be lower than the latter [21]. A second possibility is that prior CrCl values may be artifactually high as a result of serum creatinine calibration measurement problems which were not fully appreciated in prior studies. Another hypothesis is that CrCl/GFR ratio varies by patient characteristics. For example, the Shemesh paper only included patients with glomerular disease who presumably had greater proteinuria than the CRIC enrollees, although the degree of proteinuria was not reported.

Indeed some prior papers suggested that CrCl/GFR ratio increases at higher levels of proteinuria. Carrie et al. reported that the CrCl/GFR ratio was 1.22 for 10 patients with cardiac failure (and mean inulin clearance of 47 ml/min/1.73 m^2^) but it was 1.70 for 38 patients with nephrotic syndrome (and mean inulin clearance of 42 ml/min/1.73 m^2^) [22]. Branten et al. reported that in 42 patients with nephrotic syndrome (mean GFR 54 ml/min/1.73 m^2^), hypoalbuminemia was associated with more secretory clearance of creatinine and as a consequence, overestimation of GFR by endogenous CrCl is more pronounced in patients with nephrotic syndrome [4]. However, others have not observed that proteinuria influences the CrCl/GFR ratio [23-25]. Our results do not show an association between degree of proteinuria as assessed by 24-hr urine albumin and CrCl/iGFR ratio. Although the fraction of our study population with high grade proteinuria was relatively low, because of the large sample size, we still had more than 100 patients with albuminuria ≥ 3000 mg/day. We also did not find any association between hypoalbuminemia and tubular creatinine secretion, although our study did not include many patients with severe hypoalbuminemia (e.g. serum albumin <2.5 g/dl) [4].

Our study also contributes to the literature by assessing the relation between race/ethnicity and CrCl/iGFR ratio. Blacks in the U.S. population are known to have higher mean serum creatinine concentration than whites [26]. Previously, based on the relatively low CrCl/iGFR ratios (ranging from 1.01-1.21) reported out of an exclusively black CKD cohort, some investigators have speculated that there may be black-white differences in tubular handling of creatinine [6]. Our results do not support this hypothesis. We found that non-Hispanic white, and non-Hispanic black (and Hispanic) CRIC participants have similar CrCl/iGFR ratio. As alluded to before, comparing results from different publications may be confounded by differences in the exogenous filtration marker used to measure GFR and by serum creatinine calibration problems.

Our finding that use of loop diuretics was associated with higher CrCl/GFR has been noted before [27,28]. Possible mechanisms for this include reduction in GFR due to tubule-glomerular feedback [29,30] out of proportion to reduction in CrCl, hence resulting in a higher CrCl/iGFR ratio. But this rather speculative as we are not aware of any evidence for or against this hypothesis. Our report that HgA1c is associated with the CrCl/iGFR ratio is novel. However, since we screened for a large number of associations, we cannot rule out that this result is due to chance and should be evaluated in future studies.

We confirmed there was an inverse correlation between CrCl/iGFR with iGFR. This is consistent with the prior literature which showed that at progressive more severe degrees of CKD, tubular secretion of creatinine plays an increasing prominent role in clearance [3,28,31-33]. We interpreted the observation that CKD patients with higher BUN, high creatinine and lower hemoglobin had higher CrCl/iGFR ratio as being because higher BUN, high creatinine and lower hemoglobin reflect lower GFR values.

The strengths of our study are numerous and include a large, diverse sample, the wide range of GFR, the assessment of proteinuria using albuminuria, the calibration of serum creatinine measurements to an external accepted international gold standard, and the uniform assessment of GFR and CrCl across racial/ethnic groups which provide reliable information about potential racial/ethnic differences.

Limitations of our study include that iGFR and CrCl were not measured using the same UV/P blood and urine samples. Certain intrinsic renal diseases were not represented by study design such as polycystic kidney disease or myeloma kidney. We do not have information on use of trimethoprim and cimetidine, although the use of these medications are likely to be infrequent. Inaccuracies in 24-hour urine collection may have introduced random error and biased results towards the null. We did have to exclude some CRIC enrollees who were missing 24-hour albumin measurements but this should not have introduced bias given the small fraction (<6%). Our study was cross-sectional so we do not have information regarding evolution of CrCl/iGFR over time.

There are several implications of our study results. One, the CrCl/iGFR ratio is closer to 1.0 than reported by several frequently cited reports in the literature. So CrCl is closer to GFR better than the impression given by those some papers. Two, it is reassuring that factors such as degree of proteinuria (or others variables listed in Table 1) do not strongly influence CrCl/GFR ratio. Because if a multitude of factors influenced the CrCl/GFR ratio, then it would make it ever more challenging to rely on serum creatinine to estimate GFR. Three, we hope this line of work will bring fresh attention to creatinine secretion. Better understanding of determinants of creatinine secretion may have implications as a recent study reported that variants in the gene coding organic cation tranporter 2 influences both net tubular creatinine secretion and risk of end-stage renal disease [18].

To conclude, using calibrated creatinine measurements and iothalamate clearance to define GFR, we quantified the ratio of CrCl/iGFR in a large modern cohort. To our knowledge, this the largest study quantifying this important aspect of renal physiology.

## Competing interests

The authors declare that they have no competing interests.

## Authors’ contributions

YCL and CYH conceived and designed the study; ASG and CYH acquired the data; YCL and CYH drafted the manuscript; NB, EV and ASG revised it critically for important intellectual content. All authors analyzed/interpreted the data and gave final approval of the version to be published.

## Pre-publication history

The pre-publication history for this paper can be accessed here:

http://www.biomedcentral.com/1471-2369/14/268/prepub
