# Correction: Determinants of the creatinine clearance to glomerular filtration rate ratio in patients with chronic kidney disease: a cross-sectional study

**DOI:** 10.1186/1471-2369-15-85

**Published:** 2014-06-06

**Authors:** Yen-chung Lin, Nisha Bansal, Eric Vittinghoff, Alan S Go, Chi-yuan Hsu

**Affiliations:** 1 Division of Nephrology, Department of Internal Medicine, Taipei Medical University Hospital, Taipei, Taiwan; 2 Department of Internal Medicine, School of Medicine, College of Medicine, Taipei Medical University, Taipei, Taiwan; 3 Division of Nephrology, School of Medicine, University of California-San Francisco, San Francisco, CA, USA; 4 Department of Epidemiology and Biostatistics, School of Medicine, University of California-San Francisco, San Francisco, CA, USA; 5 Division of Research, Kaiser Permanente Northern California, Oakland, CA, USA

## Abstract

After the publication of our paper Lin et al. “Determinants of the creatinine clearance to glomerular filtration rate ratio in patients with chronic kidney disease: a cross-sectional study” BMC Nephrology 2013, 14:268, we became aware of errors in the manuscript arising from to a misunderstanding of serum creatinine calibration in the released Chronic Renal Insufficiency Cohort (CRIC) study data obtained from the National Institute of Diabetes and Digestive and Kidney Diseases (NIDDK) Data Repository. Specifically further multiplication by 0.95 was actually not necessary to arrive at the standardized creatinine values.

Here we present the revised results of the re-analyses along with revisions of the relevant tables. Mean CrCl/iGFR ratio should be 1.13 ± 0.46 instead of 1.19 ± 0.48. The main conclusion of the paper remain unchanged: “Contrary to what had been suggested by prior smaller studies, CrCl/GFR ratio does not vary with degree of proteinuria or race/ethnicity. The ratio is also closer to 1.0 than reported by several frequently cited reports in the literature.”

## Discussion

Under Abstract Results: it should read “Mean CrCl/iGFR ratio was 1.13 ± 0.46. There was no association between the CrCl/iGFR ratio and urine albumin (coefficient 0.10 [95% CI −0.01 - 0.21] for higest verus lowest levels of albuminuria, p = 0.07). Also, there was no association between race/ethnicity and CrCl/iGFR ratio (coefficient for non-Hispanic blacks was −0.03 [95% CI −0.08 - 0.03] compared with whites, p = 0.38).”

Under Subjects and Methods *Measures of Kidney Function*: it should read “Serum creatinine measurements were done in the CRIC central laboratory at University of Pennsylvania on the Hitachi Vitros 950 and then calibrated to the IDMS-traceable standardized creatinine [15], [16], [17].”

Under Results: it should read “The mean (±standard deviation [SD]) CrCl was 52.1 ± 25.8 mL/min/1.73 m^2^ and mean iGFR 48.0 ± 19.9 mL/min/1.73 m^2^ (Table 1). Mean CrCl/iGFR ratio was 1.13 ± 0.46 and median CrCl/iGFR ratio was 1.09 (with interquartile range [IQR] 0.88 - 1.32).” and “The only other factors associated with the CrCl/iGFR ratio were the use of loop diuretics (associated with higher CrCl/iGFR ratio of 0.08, p = 0.001)…” and “Additionally, CrCl/iGFR ratio in patients (N = 47) with serum albumin <3.0 g/dl was 1.15 ± 0.56 and that in with serum albumin ≥3.0 g/dl (N = 1260) was 1.13 ± 0.45 (p-value 0.89).”

Under Discussion: it should read: “In this well characterized cohort of CKD patients with mean measured GFR of 48 m/min/1.73 m^2^, we found that the mean CrCl/GFR ratio was 1.13.”

Tables 1−4 should read.

**Figure 1:**
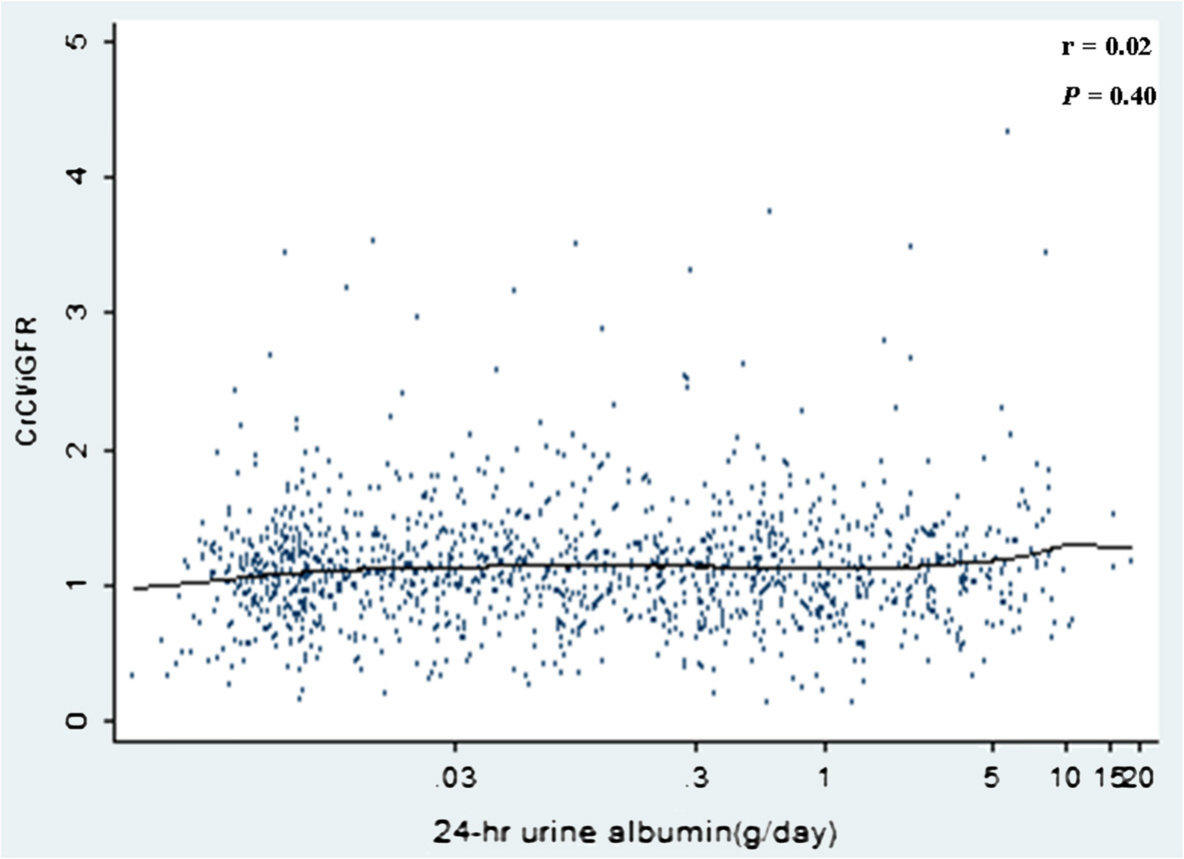
**Scatter plot with a locally weighted scatterplot smoothing line showing that 24-hrs urinary albumin is not correlated with CrCl/iGFR (rs = 0.02, *P = *** **0.40 by Spearman’s correlation test) (two outlier with CrCl/iGFR ratio of 6.40, 0.04 were omitted)” And the corrected Figure has also been included.**

**Table 1:** **Characteristics of the study population** (**N** = **1342**)

Creatinine clearance (CrCl) (mL/min/1.73 m^2^)	52.1 ± 25.8
Iothalamate measured glomerular filtration rate (iGFR) (mL/min/1.73 m^2^)	48.0 ± 19.9
CrCl/iGFR, mean ± SD	1.13 ± 0.46
CrCl/iGFR, median (25^th^-75^th^ percentile)	1.09 (0.88-1.32)
Laboratory results	
Serum creatinine (mg/dL)	1.70 ± 0.56

**Table 2:** **Creatinine clearance** (**CrCl**), **CrCl**/**iGFR**, **classified by quintiles of iothalamate**-**125 glomerular filtration rate** (**iGFR**) (**N** = **1342**)

**Quintiles of iGFR (ml/s/1.73 m**^**2**^**)**	**iGFR (ml/min/1.73 m**^**2**^**), median (IQR)**	**CrCl (ml/min/1.73 m**^**2**^**), median (IQR)**	**CrCl/iGFR, median (IQR)**
1 (N = 269)	24.8 (20.8—27.4)	30.1 (24.0—37.3)	1.26 (1.01—1.54)
2 (N = 268)	35.7 (33.0—38.5)	40.3 (32.0—48.8)	1.14 (0.89—1.40)
3 (N = 269)	45.7 (43.3—48.0)	49.8 (39.1—58.2)	1.09 (0.88—1.26)
4 (N = 268)	56.3 (53.1—59.8)	58.8 (47.6—69.4)	1.04 (0.83—1.22)
5 (N = 268)	74.7 (68.4—84.9)	77.8 (61.8—91.8)	1.00 (0.82—1.16)

**Table 3:** **The association of 24**-**hrs urinary albumin in categorical classifications and CrCl**/**iGFR ratio in the regression model** (**N** = **1342**)

	**Normal (24-hrs urine albumin < 30 mg; N = 515)**	**Microalbuminuria (24-hrs urine albumin 30 to ≦299 mg; N = 343)**	**Macroalbuminuria (24-hrs urine albumin 300 to ≦2999 mg; N = 378)**	**Nephrotic**-**range proteinuria (24-hrs urine albumin ≧3000 mg; N = 106)**
Absolute change in CrCl/iGFR (95% CI)				
Unadjusted	reference	0.05 (−0.01—0.11) *P* = 0.12	0.02 (−0.04—0.08) *P* = 0.48	0.06 (−0.03—0.16) *P* = 0.20
Multivariate adjusted^a^	reference	0.06 (−0.01—0.12) *P* = 0.08	0.04 (−0.02—0.11) *P* = 0.21	0.10 (−0.01—0.21) *P* = 0.07

*CrCl* = Creatinine clearance; *IGFR* = I^125^ Iothalamate measured glomerular filtration rate.

^a^Adjusted for age, sex, race/ethnicity, use of loop diuretics, hemoglobin A1C levels.

**Table 4:** **The association of race**/**ethnicity categories and CrCl**/**iGFR in the multivariate regression model (N = 1342)**

	**Non-Hispanic white (N = 568)**	**Non-Hispanic black (N = 494)**	**Hispanics (N = 188)**	**Others (N = 92)**
Absolute change in CrCl/iGFR ratio (95% CI)				
Unadjusted	reference	−0.03 (−0.08—0.03) *P* = 0.34	0.01(−0.07—0.08) *P* = 0.89	−0.01 (−0.12—0.09) *P* = 0.79
Multivariate adjusted^a^	reference	−0.03 (−0.08—0.03) *P* = 0.38	0.01 (−0.06—0.09) *P* = 0.72	0.01 (−0.10—0.11) *P* = 0.91

*CrCl* = Creatinine clearance; iGFR = I^125^ Iothalamate measured glomerular filtration rate.

Others = American Indian/Alaskan Native, Asian/Asian American, or Native Hawaiian/Other Pacific islander.

^a^Adjusted for age, sex, use of loop diuretics, hemoglobin A1C.

## Pre-publication history

The pre-publication history for this paper can be accessed here:

http://www.biomedcentral.com/1471-2369/15/85/prepub
